# Interaction Effects of Life Events and Hair Cortisol on Perceived Stress, Anxiety, and Depressive Symptoms Among Chinese Adolescents: Testing the Differential Susceptibility and Diathesis-Stress Models

**DOI:** 10.3389/fpsyg.2019.00297

**Published:** 2019-03-05

**Authors:** Youyun Xu, Yapeng Liu, Zheng Chen, Jing Zhang, Huihua Deng, Jiexin Gu

**Affiliations:** ^1^Key Laboratory of Child Development and Learning Science (Southeast University), Ministry of Education and Institute of Child Development and Education, Southeast University, Nanjing, China; ^2^Jiangsu Provincial Key Laboratory of Special Children’s Impairment and Intervention, Nanjing Normal University of Special Education, Nanjing, China; ^3^College of Pro-school Education, Nanjing Xiaozhuang University, Nanjing, China; ^4^College of Foreign Studies, Anhui Normal University, Wuhu, China

**Keywords:** perceived stress, anxiety, depression, hair cortisol, life events

## Abstract

The differential susceptibility model and the diathesis-stress model on the interaction effect between the individuals’ traits and environmental factors will be conducive to understand in depth whether the psychophysiological traits are the risk factors of child development. However, there is no study focusing on the activity of the hypothalamic-pituitary-adrenal (HPA) axis. We examined whether the HPA activity serves as a physiological marker of the differential susceptibility model or the diathesis-stress model by exploring the interactive effect of life events and hair cortisol on perceived stress, anxiety, and depressive symptoms among Chinese adolescents. The participants were 324 students in senior high school. They reported their psychological states with questionnaires in their first semester after a 3-month adaptation period; 2 weeks later, they provided 1-cm hair segments closest to the scalp. We measured hair cortisol concentration as a biomarker of HPA activity using high-performance liquid chromatography–tandem mass spectrometry. There was a significant interaction effect of academic events and hair cortisol on adolescents’ perceived stress, anxiety, and depression symptoms. We also observed a significant interaction between interpersonal events and hair cortisol on adolescents’ anxiety symptoms. Looking at the region of significance, proportion of interaction index, and proportion affected index, we found that adolescents with higher cortisol levels had a tendency to experience higher perceived stress and anxiety symptoms when they had high academic events scores, but lower perceived stress and anxiety symptoms when they had lower academic events scores. By contrast, adolescents with higher cortisol levels had a greater risk of experiencing high depressive symptoms only when they had higher academic events scores. Adolescents with higher cortisol levels also tended to have lower anxiety symptoms when they had higher interpersonal events scores, but greater anxiety symptoms when they had lower interpersonal events scores. These results suggested that HPA activity might serve as a biomarker of the differential susceptibility model for perceived stress and anxiety symptoms, while for depressive symptoms, it might serve as a marker of the diathesis-stress model.

## Introduction

Internalizing behavior problems, such as perceived stress, anxiety, and depressive symptoms, are prominent signals of adolescents’ degree of psychological adaptation (James, 2007). The development of such problems is considered the result of an interaction between environmental factors and adolescents’ own psychological and physiological traits; as such, researchers have begun exploring the specific moderating effects of various psychophysiological traits on the association between environmental factors and internalizing problems among adolescents (Bronfenbrenner and Morris, 2006). So far, researchers have primarily focused on the moderating effects of psychological traits (Muhtadie et al., 2013; Reuben et al., 2016). Comparatively fewer studies have examined the moderating role of physiological traits, such as monoamine oxidase A (MAO-A) gene polymorphism (Liu et al., 2017). The biological sensitivity to context theory emphasizes the importance of such physiological traits, explaining that individuals differing in biological response to stressful challenge naturally show differences in psychological adaptation (Boyce and Ellis, 2005; Zhou et al., 2016).

The hypothalamic-pituitary-adrenal (HPA) axis is a stress-sensitive nervous system responsible for the secretion of cortisol to help organs adapt to stressful events (Spiga et al., 2014; Ma et al., 2015). To date, there is no research reporting the interaction effect of environmental factors and HPA activity on internalizing problems under the differential susceptibility model and the diathesis-stress model. Therefore, determining such interaction effect will help deepen our understanding of the development of such problems in adolescents. Because life events in the family and school arguably have the strongest and most direct impact, in this study, we examined how life events and HPA activity interacted to contribute to the development of perceived stress, anxiety, and depressive symptoms among adolescents.

### Relation Between Adolescents’ Life Events and Internalizing Problems

Factors from a plethora of environments—such as the family, school, and other social environments—are known to influence the development of internalizing problems. Of these, the most direct and nearest factors are daily life events occurring in the family and school spheres (Hashmi, 2013). Stressful life events show positive associated with adolescents’ internalizing problems from moderately to highly, such as perceived stress (Ortega et al., 2012), anxiety symptoms (Lewis et al., 2012), depression symptoms (Veytia López et al., 2012), and even suicide risk (Liu and Tein, 2005). For example, Lewis et al. reported high association between and anxiety. In China, academic events (e.g.,) and interpersonal events (e.g.,) are considered the two primary sources of environmental stress experienced by adolescents (Chen et al., 2012; Xie et al., 2014). Therefore, we focused on these types of events in our study.

### Relation Between HPA Activity and Adolescents’ Internalizing Problems

Studies have found little consistency in the association between HPA activity and internalizing problems among adolescents. For example, studies have demonstrated a positive correlation (Colomina et al., 1997; Shirtcliff and Essex, 2008; Qing and Zhang, 2009; Gao et al., 2014), a negative correlation (De Bellis et al., 1996; Granger et al., 1998; Shirtcliff and Essex, 2008; Gerber et al., 2013), and no correlation (Dahl et al., 1989; Lindfors et al., 2017). The same inconsistency has been observed among children (Goodyer et al., 1991, 2001a,b; Essex et al., 2002; Smider et al., 2002; Gunnar et al., 2003).

One of the main reasons for the inconsistency is considerable gap in time between the emergency of biomarkers of HPA activity and adolescents’ internalizing problems occurring over 1 month. The HPA activity biomarkers used in most previous studies were urinary or salivary cortisol levels (Goodyer et al., 1991, 2001a,b; De Bellis et al., 1996; Essex et al., 2002; Smider et al., 2002), measured in terms of the area under curve of salivary diurnal cortisol during the daytime or within 1 day (Dickerson and Kemeny, 2004). However, these biomarkers reflect acute, or short-term, HPA activity; that is, they reflect activity over several hours or up to 1 day. Therefore, these biomarkers might not reliably reflect cortisol exposure over a longer period (e.g., 1 month), which is the period that most psychological measurements cover in their questions.

Hair cortisol levels may help address this problem. Russell et al. (2012) has suggested that this biomarker may be useful for assessing basal cortisol levels and the long-term activity of the HPA axis because it has high consistency with the average salivary cortisol level over multiple days (Zhang et al., 2018). We therefore used hair cortisol as a biomarker of HPA activity to ensure better consistency with the time span of the psychological measurements.

### Interactive Effects of HPA Activity and Life Events on Adolescents’ Internalizing Problems

Most theories and empirical studies on this topic imply that adolescents’ psychological traits can moderate the relationship between life events and internalizing problems among adolescents (Bronfenbrenner and Morris, 2006). However, as we note earlier, relatively few studies have examined the moderating role of adolescents’ physiological traits (Zheng et al., 2016). Moreover, we have not found any research specifically examining the interaction effect of HPA activity and life events on adolescent’s internalizing problems. The theoretical basis for such an association comes from research showing that children with higher cortisol levels (i.e., higher HPA activity) were more sensitive to environmental changes (e.g., a psychotherapeutic treatment) than those with lower cortisol levels (i.e., lower HPA activity) (van de Wiel et al., 2004), and were more likely to show better psychological adaptations. Additionally, Obradović et al. (2010) found that adolescents with higher cortisol reactivity showed higher prosocial behaviors (Obradović et al., 2010) and better execute functions (Obradović et al., 2016) than those with lower cortisol reactivity under less family troubles. Taken together, these studies suggest that HPA activity might interact with adolescents’ life events to predict internalizing problems.

### Diathesis-Stress and Differential Susceptibility Models

Two theoretical models have been proposed to describe how the interaction between psychophysiological traits and environmental factors (e.g., life events here) influence adolescents’ internalizing problems (Belsky and Pluess, 2009; Pluess and Belsky, 2010). The diathesis-stress model suggests that individuals with certain high-risk psychophysiological traits (e.g., negative emotionality, higher biological sensitivity, or lower HPA activity) display greater vulnerability in the face of adversity and are more liable to develop maladaptive behavioral problems, such as internalizing problems (Ellis et al., 2011). The differential susceptibility model proposes that these same individuals might also experience *better* adaptation and greater developmental plasticity when exposed to positive environmental factors (Belsky and Pluess, 2009). Some empirical studies have showed that the interaction between negative emotionality (or biological sensitivity or HPA activity) and environmental factors follows the diathesis-stress model, indicating that negative emotionality, biological sensitivity, and HPA activity are markers of the diathesis-stress model, whereas others have demonstrated that such interaction follows the differential susceptibility model. The present study examined whether the interactions between the HPA activity and life events follow these two models.

### The Present Study

Regarding the interaction between physiological traits and environmental factors, previous studies have typically focused on genotypes, such as MAO-A gene polymorphism or the serotonin transporter-linked polymorphic region (5-HTTLPR) (Liu et al., 2018). HPA activity, as noted earlier, reflects a biological sensitivity of individuals’ response to stress (Ellis et al., 2005) and might act as an intermediary between gene expressions and internalizing problems (Ellis et al., 2011). Although genes might regulate the specific pattern of HPA activity, HPA activity and gene polymorphisms may differ greatly in how they interact with environmental factors on. Thus, it is unclear if the results from research on gene polymorphism can be generalized to HPA activity.

Additionally, research on the interactive effects of physiological traits and environmental factors has mostly been conducted in developed countries or in regions with western individualistic cultures, such as North America or Western Europe. Individualistic cultures differ markedly from collectivistic cultures such as China, and cultural beliefs and values may regulate patterns of HPA activity as well as the development of adolescents’ internalizing problems (Chen, 2012). Accordingly, the findings from western cultures might not be generalized to adolescents in China.

This study examined the interactive effects of HPA activity and life events on perceived stress, anxiety, and depressive symptoms among Chinese adolescents. Cortisol concentration in a 1-cm hair sample was utilized as the biomarker of basal HPA axis activity over 1 month, ensuring that the cortisol measurement matched the time span for the measurement of psychological variables. Based on the above background, we expected that hair cortisol and life events would interact to predict adolescents’ internalizing problems. Drawing on the diathesis-stress and differential susceptibility models, we proposed two alternative hypotheses. From the perspective of the diathesis-stress model, we hypothesized that adolescents with higher cortisol levels perceive higher stress and experience greater anxiety and depressive symptoms than do those with lower cortisol levels when they encounter more stressful life events. However, the two cortisol-level groups would not differ under less stressful life events. By contrast, from the perspective of the differential susceptibility model, we hypothesized that adolescents with higher cortisol levels would develop more negative outcomes than would those with lower cortisol levels when experiencing more stressful life events, whereas they would how better outcomes when experiencing less stressful events.

## Materials and Methods

### Participants

Four hundred sixty students from an ordinary senior high school in Nanjing city, China participated in the present study. All students were of Han ethnicity. Among them, 29 students were excluded because they did not meet the inclusion criteria for hair collection, which were as follows: (a) hair length longer than 1 cm; (b) physically and mentally healthy and no chronic diseases; (c) no experience of smoking or drinking, not currently taking medicine, and did not have dyed or bleached hair (as this could influence the concentration of hair cortisol; Wosu et al., 2015); and (d) no experience of major stressful events, such as the death of a family member, traffic accident, and parental divorce, over the past year. Ultimately, 431 students completed the questionnaires. Of these, 324 students (mean age: 15.35 ± 0.41 years; age range: 15–16 years), including 133 boys and 191 girls, provided hair samples. We observed no differences in gender distribution, age, life events, or psychological adaptation (*p*s > 0.05) between the participants who did not provide hair samples and those who did.

Prior to recruitment, we obtained verbal consent from the students and the teachers in charge of them, and then obtained written consent from students’ guardians. All the participants then provided written consent form for the questionnaire survey, while 324 participants provided their consent for the hair collection. The present study followed the Declaration of Helsinki and was approved by the Health Science Research Ethics Board of Southeast University, China.

### Procedures

The participants were recruited during their first semester in late November after a 3-month period of adaptation to senior high school. After signing the written consent form, they reported their demographic information, life events, and psychological outcomes over the past 1 month using the questionnaire provided to them.

Because about 1–3 mm of the hair shaft is embedded deep in the scalp and 1–2 mm of which is too close to the scalp to be cut with scissors, considering that the average hair growth rate is ∼1 cm per month (Pragst and Balikova, 2006), two weeks later, we collected hair strands in the posterior vertex region, cutting them as close to the scalp as possible to ensure accurate measurement of cortisol concentration.

### Measures

#### Academic and Interpersonal Events

Academic and interpersonal events were measured using the academic and interpersonal subscales of the Chinese-version Life Events Rating Scale for Adolescents (Liu et al., 1997). This scale comprises 27 items, each of which assesses adolescents’ experience of a negative life event that could lead to a psychological response and to what degree the event has affected the adolescents. The 27 items are grouped into 6 subscales: academic events, interpersonal events, getting disciplined, deprivation, health adaptation, and others. The academic and interpersonal subscales each contain five items rated on a 6-point scale (1–6). We averaged the scores of each subscale for the analysis, with higher scores indicating greater stress from negative life events (Liu et al., 1997). The Cronbach’s *α* coefficients of the academic and interpersonal subscales were 0.73 and 0.72, respectively.

#### Perceived Stress

Perceived stress was measured with the Perceived Stress Scale designed by Cohen et al. (1983) and adapted into Chinese by Yang and Huang (2003). The scale consists of 14 items measuring individuals’ feeling of nervousness and a loss of control, each of which is scored on a 5-point scale ranging from 0 to 4. The sum of the item scores was calculated to serve as a total score, with higher scores indicating greater perceived stress. In this study, the Cronbach’s α coefficient of this measure was 0.89.

### Anxiety Symptoms

Anxiety symptoms were assessed with the state anxiety subscale of the State-Trait Anxiety Inventory (Spielberger and Gorsuch, 1983). This subscale comprises 20 items describing adolescents’ emotional experience of anxiety in response to different situations, such as fear and nervousness. Items are scored on a 4-point scale ranging from 1 to 4. The inventory shows relatively high reliability and validity in China (Fu, 1997). In this study, the Cronbach’s α coefficient was 0.76. The total score (the sum of the item scores) was used as an index of anxiety symptoms.

#### Depressive Symptoms

Depressive symptoms were measured using the Zung Self-Rating Depression Scale (Zung et al., 1965). This scale consists of 20 items describing relevant symptoms of depression. Each item is scored on a 4-point scale ranging from 1 to 4. The total score of the items was calculated, with higher scores indicating more severe depressive symptoms. The scale has relatively high reliability and validity in China (Feng et al., 2005). In this study, the Cronbach’s α coefficient of this scale was 0.76.

#### Hair Cortisol Measurement

Before analysis, the hair samples were cut into 1-cm segments. Those segments closest to the scalp were used to measure average cortisol concentration over the past month. The hair pieces were washed with methane and then dried in shade twice. Then the 20 mg clean sections were finely cut into pieces and incubated in 0.9 methanol for 1 day. Hundred μl Cortisol-d4 as internal standard was added at 10 ng/ml in the incubation. After that, the incubated solution was centrifuged at 10,000 rpm for 1 min. Afterward a 400 μl supernatant was poured into another dry tube and then dried with nitrogen at 40° centigrade. Finally, the dried sample was redissolved in 80 μl methane for further analysis. The measurement of hair cortisol was conducted using high-performance liquid chromatography–tandem mass spectrometry; the assay method is described in detail elsewhere (Qi et al., 2016). The assay method showed good performance, such as good linearity in the range of 1.0–100.0 pg/mg, lower limits of detection and quantification at 0.5 and 1.0 pg/mg, good recovery at 99.2 ± 8.3% (2 pg/mg) and 103.1 ± 7.3% (20 pg/mg), and intra-day and inter-day coefficients of variation less than 10% (5.4 and 8.2% for 2 pg/mg; 4.3 and 7.5% for 20 pg/mg), respectively.

### Statistical Analysis

SPSS Statistics 20.0 for Windows was used to conduct a test of data normality, independent samples *t*-tests, correlation analysis, and regression analysis. The data normality was examined with the Kolmogorov–Smirnov (K–S) test. Normally distributed data are presented as means (*M) ±* standard deviations (*SD*), while non-normally distributed data are presented as medians and ranges and were log-transformed for subsequent statistical analyses. Hierarchical multiple regression analysis was used to investigate the interaction effects of life events and hair cortisol on juvenile psychological adaptation. Simple slope analysis was conducted as suggested by Aiken and West (1994) to further explore how hair cortisol moderates the relationship between life events and psychological adaptation.

To determine whether the results followed the differential susceptibility model or the diathesis-stress model, we used the approach of regions of significance (RoS) with the probing interaction procedure as developed by Hayes and Matthes (2009). This involved probing the interactions between -2*SD* and +2*SD* of the mean of the independent variables (i.e., life events here). Additionally, the proportion of interaction index (PoI) and the proportion affected index (PA) were examined as straightforward markers (Roisman et al., 2012). The PoI index is defined as the ratio of better outcomes over the sum of all outcomes (better and worse) for the higher and lower cortisol level groups when the independent variable was bounded by ± 2*SD* of the mean. The PA index, on the other hand, is defined as the proportion of the population that is differentially affected by hair cortisol level—that is, the population that falls above the crossover point of the independent variable (i.e., the point at which the regression lines between independent variable and dependent variable cross over; Roisman et al., 2012). Typically, the PoI and PA indices will range from 0.00 to 0.50.

If either the upper and lower limits of the RoS exceeded the ±2*SD* range of the mean of the independent variable, while the other remains bounded to that range, the interaction should be interpreted as strong evidence of the diathesis-stress model. If both limits of the RoS were bounded to the ±2*SD* range, then the interaction would be interpreted as evidence for the differential susceptibility model. Similarly, if the PoI and PA indices are close to 0.50, the interaction would be in favor of the differential susceptibility model; if their values are less than 0.16, the interaction is not likely to support the differential susceptibility model; if their values are closer to 0.00, the interaction would be in support of the diathesis-stress model (Roisman et al., 2012).

## Results

### Descriptive Results and Correlation Analysis

Table 1 shows the descriptive statistics of all variables and their intercorrelations. Both academic events and interpersonal events were significantly and positively associated with perceived stress, anxiety, and depression symptoms (*p*s < 0.001). Hair cortisol was significantly and positively associated with perceived stress and depressive symptoms (*p*s < 0.05), and marginally positively associated with academic events (*p* = 0.06) and interpersonal events (*p* < 0.05). Additionally, male students had significantly lower depressive symptoms (*t*_322_= -3.01, *p* < 0.01) and higher hair cortisol levels (*t*_322_= 3.92, *p* < 0.001) than did female students, but there were no differences between them in perceived stress scores and anxiety symptoms (*p*s > 0.05).

**Table 1:** Descriptive statistics and correlation analysis for adolescents’ life events, hair cortisol, and internalizing problems.

	*M*	*SD*	1	2	3	4	5	6
(1) Academic events	3.56	0.93	-	0.42^∗∗∗^	0.11^+^	0.429^∗∗∗^	0.41^∗∗∗^	0.41^∗∗∗^
(2) Interpersonal events	2.67	0.90		-	0.14^∗^	0.27^∗∗∗^	0.30^∗∗∗^	0.31^∗∗∗^
(3) Hair cortisol (pg/mg)^a^	6.6	2.0–29.7			-	0.11^∗^	0.04	0.12^∗^
(4) Perceived stress	25.03	7.55				-	0.67^∗∗∗^	0.68^∗∗∗^
(5) Anxiety symptoms	42.02	10.14					-	0.72^∗∗∗^
(6) Depressive symptoms	41.14	6.79						-


*^+^*p* < 0.10, ^∗^*p* < 0.05, ^∗∗^*p* < 0.01, ^∗∗∗^*p* < 0.001. ^*a*^Hair cortisol concentrations were presented as medians and ranges because they were non-normally distributed (*p* < 0.001) as examined by the Kolmogorov–Smirnov test. They were log-transformed for the correlation analysis. The log-transformed cortisol concentrations (1.10 ± 0.44) were normally distributed (*p* = 0.193) as examined by the Kolmogorov–Smirnov test because the log transformation effectively reduced the skewness and kurtosis.*

### Regression Analysis

A series of hierarchical multiple linear regression analyses were conducted to examine the interaction between academic or interpersonal events and hair cortisol in predicting perceived stress scores, anxiety, and depression symptoms. Adolescents’ gender was entered as a control variable in the first step, while academic events, interpersonal events, and hair cortisol were entered in the second step. In the final step, the interactions between life events and hair cortisol were entered. Following Aiken and West (1994), academic events, interpersonal events, and hair cortisol were centered in the analyses.

As shown in Table 2, academic events positively predicted adolescents’ perceived stress, anxiety, and depression symptoms (*p*s < 0.001), and interpersonal events positively predicted adolescents’ anxiety and depressive symptoms (*ps* < 0.001). The interaction between academic events and hair cortisol also significantly predicted adolescents’ perceived stress, anxiety, and depressive symptoms (*p* < 0.01, *p* < 0.05, and *p* < 0.01, respectively), while the interaction between interpersonal events and hair cortisol significantly predicted adolescents’ anxiety symptoms (*p* < 0.01).

**Table 2:** Effects of life events, hair cortisol, and their interactions in predicting perceived stress scores, anxiety, and depressive symptoms among Chinese adolescents.

Predictor	Perceived stress scores				Anxiety symptoms				Depression symptoms			
	Δ*R*^2^	*B*	*SE*	*β*	Δ*R*^2^	*B*	*SE*	*β*	Δ*R*^2^	*B*	*SE*	*β*
Layer 1	0.01				0.001				0.02^∗^			
Gender		-1.10	0.84	-0.07		-0.68	1.12	-0.03		-1.87	0.75	-0.14^∗^
Layer 2	0.22^∗∗∗^				0.23^∗∗∗^				0.21^∗∗∗^			
AAE^a^		3.41	0.47	0.41^∗∗∗^		4.15	0.63	0.37^∗∗∗^		2.38	0.43	0.31^∗∗∗^
IPE^a^		0.77	0.48	0.09		1.90	0.64	0.17^∗∗^		1.44	0.43	0.19^∗∗∗^
HCC^a^		1.32	0.86	0.08		-0.21	1.13	-0.01		1.45	0.77	0.10^+^
Layer 3	0.02^∗^				0.02^∗^				0.02^∗^			
AAE × HCC ^b^		3.41	1.09	0.17^∗∗^		3.27	1.44	0.13^∗^		2.82	0.98	0.17^∗∗^
IPE × HCC^b^		-1.40	1.09	-0.08		-4.16	1.45	-0.17^∗∗^		-1.73	0.98	-0.10


*^+^*p* < 0.10, ^∗^*p* < 0.05, ^∗∗^*p* < 0.01, ^∗∗∗^*p* < 0.001. ^*a*^AAE, academic event; IPE, interpersonal event; HCC, hair cortisol concentration. ^*b*^The interaction between academic or interpersonal events and hair cortisol concentration. Collinearity diagnostics revealed that the tolerance was higher than 0.2 and the variance inflation factor (VIF) was less than 5 for each predictor in the regression analyses, indicating no multicollinearity problems (Fox, 1991).*

### The Moderating Influence of Hair Cortisol on the Association Between Life Events and Psychological Adaptation

**FIGURE 1:**
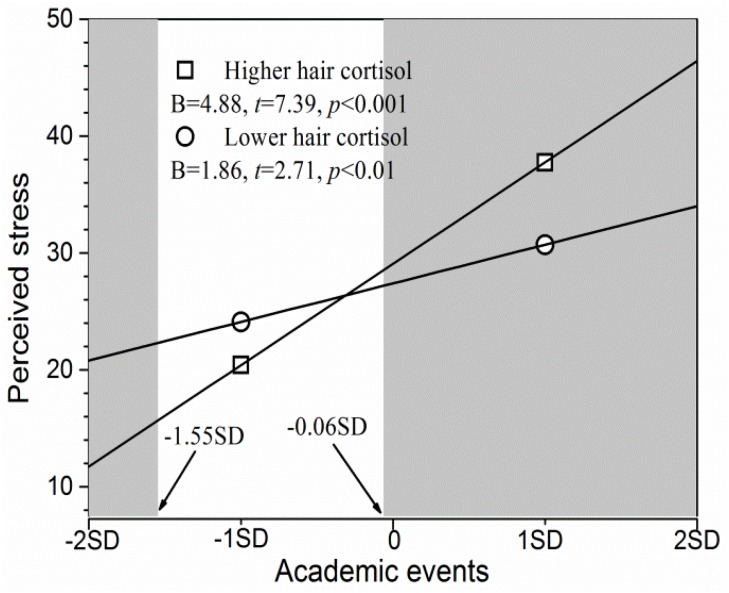
Hair cortisol as a moderator of the association between academic events and perceived stress. The shaded area represents the region of significance. Inserted is the simple slope (*B*).

As shown in Figure 1, simple slope analysis revealed that academic events significantly and positively predicted perceived stress scores for adolescents at both higher and lower cortisol levels (*p*s < 0.05), although the effect was stronger for individuals with higher cortisol levels relative to those with lower cortisol levels (*B* = 4.88 vs. *B* = 1.86). The RoS approach revealed that adolescents with higher cortisol levels had significantly higher perceived stress scores than did those with lower cortisol levels when the academic events scores were more than -0.06 *SD* from the mean (see the right shaded area in Figure 1), and had significantly lower perceived stress scores when academic event scores were less than -1.55 *SD* from the mean (the left shaded area in Figure 1). However, the two groups did not differ markedly in terms of perceived stress when academic events scores ranged from -1.55 to -0.06 *SD* of the mean. Furthermore, the PoI index was 0.22 and the PA index was 0.71. Taken together, these results seem to support the differential susceptibility model as opposed to the diathesis-stress model.

**FIGURE 2:**
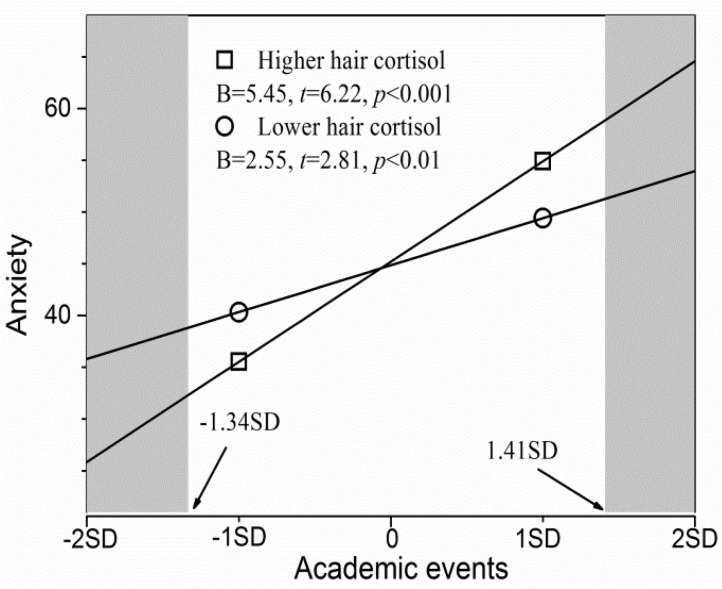
Hair cortisol is a moderator of the association between academic events and anxiety symptoms. The shaded area represents the region of significance. Inserted is the simple slope (*B*).

As for the association between academic events and anxiety symptoms, similar results were observed in the simple slope test. As shown in Figure 2, academic events significantly and positively predicted anxiety symptoms at both higher and lower cortisol levels (*p*s < 0.05), but the effect was stronger for the former group relative to the latter (*B* = 5.45 vs. *B* = 2.55). These findings indicate that hair cortisol levels influence the strength of the association between the academic events and anxiety symptoms in both positive and negative contexts. The RoS approach revealed that adolescents with higher cortisol levels had significantly greater anxiety symptoms than did those with lower cortisol levels when academic events scores were more than 1.41 *SD* of the mean (the right shaded area in Figure 2); however, they had significantly less anxiety symptoms when the academic events scores were less than -1.34 *SD* of the mean (the left shaded area in Figure 2). The two groups did not differ in terms of anxiety symptoms when the academic events ranged from -1.34 to 1.41 *SD* of the mean. Furthermore, the PoI index was 0.43 and the PA index was 0.55. These results again supported the differential susceptibility model rather than the diathesis-stress model.

**FIGURE 3:**
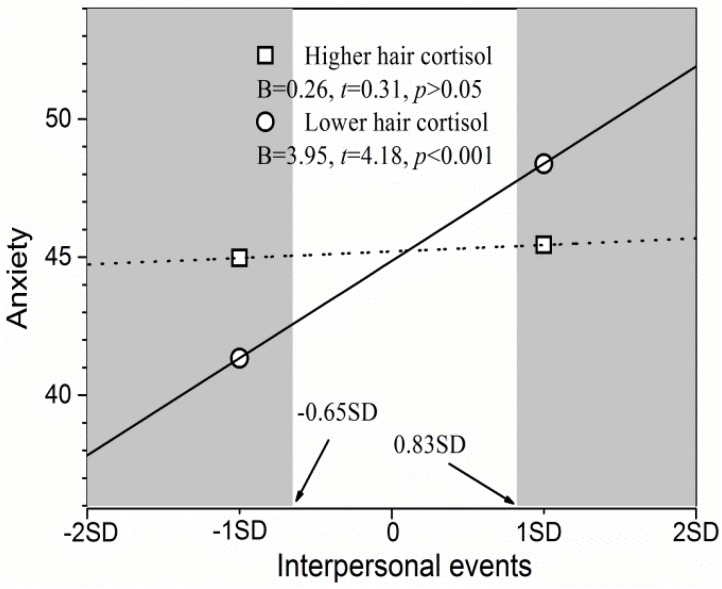
Hair cortisol as a moderator of the association between interpersonal events and anxiety symptoms. The shaded area represents the region of significance. Inserted is the simple slope (*B*).

As for the association between interpersonal events and anxiety symptoms, simple slope tests (see Figure 3) revealed that the association was significant for adolescents at lower cortisol levels (*p* < 0.001), but not significant at higher cortisol levels (*p* > 0.05). The RoS approach revealed that adolescents with higher cortisol levels had significantly lower anxiety symptoms than did those with lower cortisol levels when the interpersonal events scores were more than 0.83 *SD* of the mean (the right shaded area in Figure 3). Furthermore, they had significantly higher anxiety symptoms when interpersonal events scores were less than -0.65 *SD* of the mean (the left shaded area in Figure 3). However, the two groups did not differ from each other in terms of anxiety symptoms when interpersonal events ranged from -0.65 to 0.83 *SD* of the mean. Furthermore, the PoI index was 0.55 and PA index was 0.46. Therefore, the differential susceptibility model was once again supported.

**FIGURE 4:**
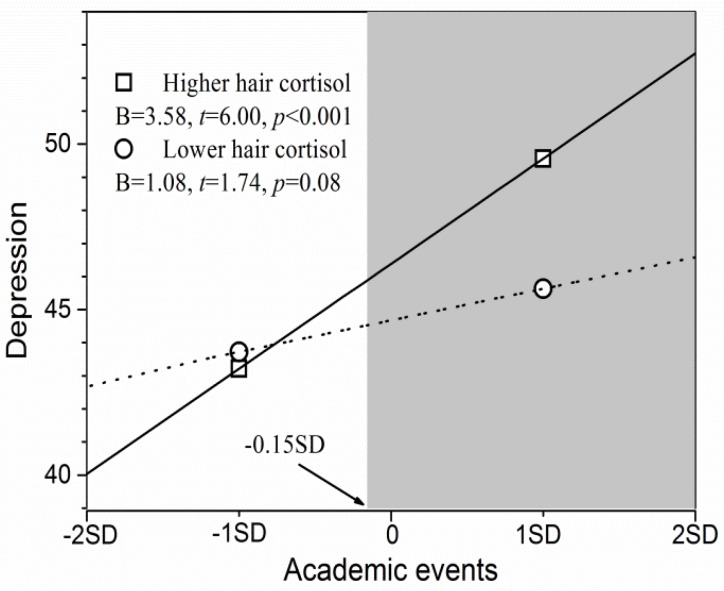
Hair cortisol as a moderator of the association between academic events and depressive symptoms. The shaded area represents the region of significance. Inserted is the simple slope (*B*).

Finally, regarding the association between academic events and depression symptoms, simple slope tests revealed that the prediction was significant for adolescents with higher cortisol levels in Figure 4 (*B* = 3.58, *p* < 0.001), but non-significant for those with lower cortisol levels (*p* > 0.05). The RoS approach revealed that adolescents with higher cortisol levels had significantly greater depression symptoms than did those with lower cortisol levels when the academic events scores were more than -0.15 *SD* of the mean (the shaded area in Figure 4), and had lower depression symptoms than did those with lower cortisol levels when the academic events scores were less than -2.13 *SD* of the mean (note that this is beyond -2 *SD* of the mean). Notably, the two groups did not differ when academic events scores were less than -0.15 *SD* of the mean. Furthermore, the PoI index was 0.16 and the PA index was 0.25. Taken together, these results suggest strong support for the diathesis-stress model.

## Discussion

The results of the study indicated that HPA activity interacted with academic events in predicting perceived stress, anxiety symptoms, and depressive symptoms, while interpersonal events interacted with HPA activity to predict anxiety symptoms among Chinese adolescents in senior high school. Interestingly, HPA activity showed greater variation in their interaction patterns depending on the outcome—following both the differential susceptibility and diathesis-stress models—when compared with genotypes, most of which showed a single interaction pattern (e.g., the diathesis-stress model; Liu et al., 2017, 2018). The findings provide new evidence for the extension of the differential susceptibility and diathesis-stress models to other physiological traits closely related to genotypes.

Adolescents with higher hair cortisol levels not only were more likely to exhibit higher perceived stress and anxiety symptoms than were those with lower hair cortisol levels when they have experienced more stressful academic events, but also were more likely to show lower perceived stress and anxiety symptoms when encountering less stressful academic events. Similarly, adolescents with lower hair cortisol levels tended to experience greater anxiety symptoms than did those with higher hair cortisol levels under more stressful interpersonal events, but lower anxiety symptoms under less stressful interpersonal events. Taking these results together with previous findings on the interaction between salivary cortisol stress reactivity and family factors (e.g., adversity and income) in predicting children’s socioemotional behavior and cognitive development (Obradović et al., 2010, 2016), the differential susceptibility model appears to be supported. It indicated that the HPA activity is the plasticity factor for Chinese adolescents’ perceived stress and anxiety symptoms. In other words, higher HPA activity strengthens association of academic events with perceived stress and anxiety symptoms and weakens association of interpersonal events with anxiety symptoms compared with lower HPA activity. We discuss each of these findings in turn below.

The moderating effect of higher hair cortisol level (i.e., higher HPA activity) on the association of academic events with perceived stress and anxiety symptoms is perhaps explained by the fact that individuals with high basal cortisol levels are in a relatively higher stress-related arousal state. This may make them somewhat more sensitive to stressful events and perceive higher stress as demonstrated in the previous studies (Karlén et al., 2011; Huang et al., 2014; Staufenbiel et al., 2014). In the present study, adolescents with higher basal cortisol are also perhaps more easily affected by external environmental factors, such as stressful academic events which are arguably the most common negative life events among Chinese senior high school students. This explanation is supported by previous studies indicating that salivary cortisol stress reactivity moderates the relation between external factors and children’s socioemotional behavior and cognitive development (van de Wiel et al., 2004; Obradović et al., 2010, 2016). van de Wiel et al. (2004) found that children with high cortisol responsivity tended to improve more in externalizing behavioral problems than did those with low cortisol responsivity after a 9-month psychotherapeutic treatment. Obradović et al. (2010) further noted that children with high cortisol reactivity were more likely to demonstrate maladaptive outcomes (e.g., lower prosocial behaviors and executive function performance) under conditions of high family adversity (e.g., high parenting overload and family financial stress), but more adaptive outcomes under conditions of lower family adversity. We also found that lower hair cortisol (i.e., lower HPA activities) influenced adolescents’ anxiety symptoms under stressful interpersonal events, unlike under stressful academic events. This might be because academic events, as the most stressful events for Chinese high school adolescents, exert stronger and more sustained effects on students when compared to other life events. Nowadays, the examination-oriented education system makes academic achievements a collective obsession among Chinese parents and teachers. This deep-rooted tradition has made academic events the greatest chronic stressors for Chinese adolescents (as our findings demonstrated, with adolescents experiencing significantly higher stress from academic events than from interpersonal events). Moreover, the majority of academic events are objective events that pervade adolescents’ lives and have a stable and sustained effect. In contrast, interpersonal events are somewhat more subjective and possess a more volatile and temporary nature; that is, they do not necessarily persist over long periods. Individuals with higher basal cortisol levels may therefore experience relatively greater fatigue in trying to cope with the sustained effects of stressful academic events. As a result, they may have reduced ability to cope with occasional interpersonal events, as suggested by the allostasis theory and resource conservation theory (Mcewen and Stellar, 1993; Mcewen, 2003), making them more insensitive to these events. By contrast, individuals with lower basal cortisol levels would have relatively more intact physical and mental resources due to an insensitivity to academic events. This could give them excess physical and mental resources to perceive interpersonal events, thereby increasing their anxiety symptoms.

Higher hair cortisol levels (i.e., higher HPA activities) was a risk factor for depressive symptoms under stressful academic events. Specifically, individuals with higher hair cortisol levels tended to be more depressed than did those with lower cortisol levels under more stressful academic events, but there was no difference between the groups under less stressful academic events (i.e., both groups showed lower depressive symptoms). This is the only finding that supported the diathesis-stress model in this study. Thus, the interaction between HPA activity and academic events showed differing patterns for anxiety and depressive symptoms, even though both types of symptoms are considered internalizing problems. This is perhaps because the activation and regulation mechanism of the HPA axis differs between anxiety and depression. Previous studies have shown that patients with generalized anxiety disorders tend to have lower hair cortisol levels than do healthy individuals, which might result from excess activation of the HPA axis. Such excess activation could induce the excess sensitivity of HPA negative feedback and eventually lead to impaired cortisol levels (Wester and van Rossum, 2015). However, individuals with depressive disorder show relatively higher cortisol levels (Vreeburg et al., 2010; Wester and van Rossum, 2015), which might result from the reduced sensitivity of the negative feedback circuit of the HPA axis or impaired corticosteroid receptor signaling observed in patients with major depression (Holsboer, 2000), following sustained activation of the HPA axis. Moreover, individuals with major depressive disorder take longer to recover to their basal cortisol level (Burke et al., 2005; Vreeburg et al., 2010) after the activation of their HPA axis to cope with external stress. Therefore, higher HPA activity may be a risk factor for individuals with greater depressive symptoms under more stressful academic events, but not under less stressful academic events.

This study has some limitations. The present study did not consider the influence of socioeconomic status except for family income. Although this study didn’t find significant association between family income and dependent variables, the future research needs to investigate the influence of socioeconomic status.

Overall, higher HPA activity is associated with greater perceived stress and anxiety symptoms under stressful academic events, while lower HPA activity is associated with higher anxiety symptoms under stressful interpersonal events. These results imply that moderate stress is more beneficial than is extremely high or low stress. Moderate stress can help students maintain an appropriate level of physiological and mental arousal, which in turn may optimally benefit performance (as predicted by the Yerkes-Dodson law; Broadhurst, 1957). In fact, there is an inverted U relationship between stress and performance where both too high and too low stress would decrease performance. It is therefore necessary to build a context where academic achievement is an important, but not a dominant, focus in China. This will help students improve their mental and physical health and academic performance. As for the students who are struggling with learning difficulties, educators should be more concerned about their HPA axes of higher activity and protect them from being crushed by high stress.

## Author Contributions

All authors listed have made a substantial, direct and intellectual contribution to the work, and approved it for publication.

## Conflict of Interest Statement

The authors declare that the research was conducted in the absence of any commercial or financial relationships that could be construed as a potential conflict of interest.
